# Surgical treatment for bronhial carcinoid tumors prognostic factors for long-term outcome

**DOI:** 10.1186/1749-8090-8-S1-O231

**Published:** 2013-09-11

**Authors:** D Petrov, D Marinova, E Goranov, Y Slavova, G Yankov

**Affiliations:** 1 Thoracic Surgery, Saint Sophia University Hospital for Pulmonary Diseases, Medical University, Sofia, Bulgaria; 2 Pneumology, Saint Sophia University Hospital for Pulmonary Diseases, Medical University, Sofia, Bulgaria

## Background

The aim of study was to study the prognostic factors after surgery with curative intend for pulmonary carcinoid tumors - typical carcinoid (TCs) and atypical carcinoid (AtCs).

## Methods

Surgically resected specimen (men n=59;women n=72, age 48±15) from 111 TC (84.7%) and 20 AtC (15.3%) patients were studied. The histological type, T-status, N-status (TC - 94 N0, 17 N1/2; AtC - 16 N0, 4 N1), pTNM stage (TC-I n=81, II n=17, III+IV=13; AtC-I n=12, II-IV n=8), surgery (55 simple lobectomies, 16 bilobectomies, 24 pneumonectomies (4 extended), 15 segmentectomies, 16 sleeve lobectomy (in 2 of them combined with sleeve resection of pulmonary artery), 5 sleeve resection of main bronchus), and immunohistochemical expression (on 64 TCs and 13 AtCs) of p-HspB5, Hsp27 and mTOR were evaluated. Kaplan-Meier, Wilcoxon, Cox regression analyses were the statistical methods used.

## Results

The overall 5-, 10- and 15-year survival for TCs were 84%, 70% and 62% respectively; and for AtCs–53% and 0%. The difference in mean survival time between TCs (14 years) and AtCs (7 years) was significant (p=0.004).The median survival time was significantly longer in N0 status (N0-213 months,N1/2-54,p=0.002), in I and II pTNM status (I/II-213 months, III/IV-27,p=0.020) and in cases positive for mTOR (p=0.035). T status, p-HspB5, Hsp27 expression were not statistically significant factors for survival (p>0.05).The Cox analysis confirmed the prognostic significance of histology type (HR 3.32;p=0.007), N status (HR 3.51;p=0.014) and pTNM stage (HR 11.27;p<0.001).

## Conclusion

Postoperative survival is significantly related to the histology type, N status, pTNM stage and mTOR expression.

